# Adolescent with Foot Pain

**DOI:** 10.5811/westjem.2016.6.30142

**Published:** 2016-07-19

**Authors:** Nicole Mansfield, Sarab Sodhi, Richard Pescatore, Andrew Nyce

**Affiliations:** Cooper University Hospital, Department of Emergency Medicine, Camden, New Jersey

A previously healthy 14-year-old female presented to the emergency department because of a left foot injury. She reported “twisting” her foot while walking. She complained of left foot pain, but was able to ambulate with a limp. Physical exam was significant for soft tissue edema and isolated tenderness to the dorsum of the left foot in the region of the cuboid. She had full range of motion at the ankle joint without pain or tenderness and was neurovascularly intact. Radiographs were obtained and revealed cuboid subluxation (Figure).

## DIAGNOSIS

### Cuboid subluxation

Cuboid subluxation is generally diagnosed clinically, based on history and physical exam, and definitive radiographic evidence is rare. Injury presentation is varied, but case reports often describe pain isolated over the cuboid following a forceful inversion of the foot while bearing weight. Physical exam typically reveals tenderness over the cuboid. Because of normal variations between the cuboid and its surrounding structures, traditional radiographs, computerized tomography, and magnetic resonance imaging are generally non-diagnostic for this injury.^1^–^8^ Additionally, midtarsal joint abnormalities that cause pain while weight bearing may be missed on non-weight bearing films.^2^ Imaging, however, is often ordered even when suspicion for cuboid subluxation is high to rule out other causes of foot pain.

In the case image above (Figure), there is lateral displacement of the cuboid relative to the base of the fourth metatarsal (arrows). A good rule-of-thumb and highly suggestive radiographic finding of cuboid subluxation is a disturbed relationship between the medial-cuboid and its articulation with the fourth metatarsal.

Treatment may require manual reduction and responds well to conservative physiotherapy management. Our patient was fitted with a controlled ankle motion (CAM) walking boot and was given crutches. She was referred to the orthopedic surgery clinic for further management.

## Figures and Tables

**Figure:**
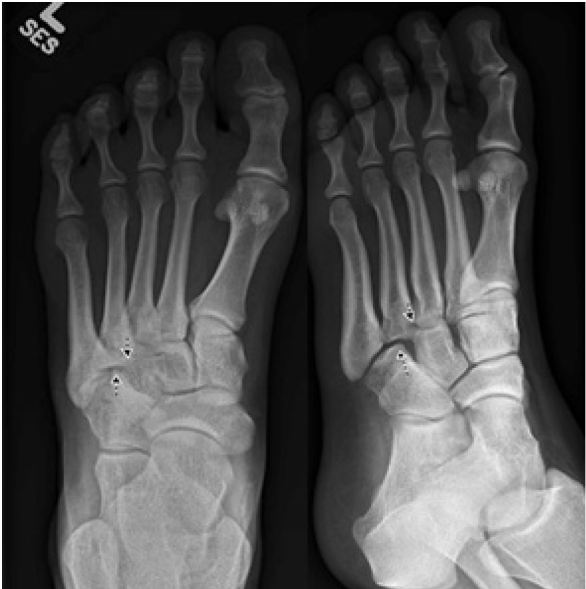
Anterior-posterior (left) and oblique (right) radiograph of the left foot with lateral displacement of the cuboid relative to the base of the fourth metatarsal (arrows).
